# Endotoxin Binding by Sevelamer: Potential Impact on Nutritional Status

**DOI:** 10.1155/2013/954956

**Published:** 2013-01-17

**Authors:** Natsuki Kubotera, Alexander J. Prokopienko, Adinoyi O. Garba, Amy Barton Pai

**Affiliations:** ^1^Department of Pharmacy Practice, Albany College of Pharmacy and Health Sciences, 106 New Scotland Avenue, Albany, NY 12208, USA; ^2^Albany Nephrology Pharmacy Group (ANephRx), Albany College of Pharmacy and Health Sciences, 106 New Scotland Avenue, O'Brien 232, Albany, NY 12208, USA

## Abstract

Patients on hemodialysis (HD) have a high burden of chronic inflammation induced associated with multiple comorbidities including poor nutritional status. Endotoxin (ET) is a Gram-negative bacterial cell wall component and a potent stimulus for innate immune system activation leading to the transcription of proinflammatory cytokines (e.g., IL-1, IL-6, and TNF**α**) that adversely affect protein metabolism and nutrition. Several cross-sectional observational studies have found that elevated serum ET concentrations in hemodialysis patients are associated with lower serum albumin, higher proinflammatory cytokine, and C-reactive protein concentrations. Possible sources of ET in the systemic circulation are bacterial translocation from the gastrointestinal tract and iron supplementation, potentially leading to intestinal bacterial overgrowth. Sevelamer is a nonabsorbable hydrogel approved for use as a phosphate binder in HD patients. Reductions in serum ET concentrations in hemodialysis patients have been observed with sevelamer therapy in observational studies and the few published interventional studies. Reduction of ET concentrations was associated with concomitant reductions in TNF**α**, IL-6, and CRP and improvement in serum albumin in the majority of these small studies. Additional studies are needed to evaluate the potential effects of sevelamer treatment on nutritional status in chronic kidney disease (CKD) patients with elevated ET.

## 1. Introduction

Proinflammatory cytokines such as IL-1, IL-6, and TNF-*α* and the anti-inflammatory cytokine IL-10 are elevated in hemodialysis (HD) patients [1]. Several factors are linked with the Proinflammatory state in end-stage renal disease (ESRD) patients on dialysis including nutritional status, diabetes, hypertension, sepsis, and biocompatibility with dialysis membranes [1]. Poor nutritional status is a vexing clinical problem that occurs in up to 50% of ESRD patients on hemodialysis and is associated with increased mortality [2]. Documented appetite loss in ESRD patients is associated with higher mortality rates [3]. Albumin is a negative acute phase reactant, and low serum albumin concentrations are associated with elevated markers of inflammation including IL-6, CRP, and TNF-*α* [4–6]. ESRD patients on HD exhibit increased protein catabolism profiles and greater skeletal muscle breakdown that is correlated with reduced serum albumin concentrations [5, 7, 8]. This loss of lean body mass in concert with chronic inflammation has been identified as a major risk factor for cardiac heart failure in ESRD patients [9, 10]. 

Endotoxin (ET) or lipopolysaccharide (LPS) is a major glycolipid component of Gram-negative bacteria cell wall and a potent inducer of inflammatory cytokine production via signaling pathway initiated by stimulating the toll-like receptor 4 (TLR4) [11]. Patients receiving hemodialysis can also be exposed to ET through dialysate. Currently, the Association for the Advancement of Medical Instrumentation endorses an action level for an ET concentration of 0.125 ET units/mL (EU/mL) for water used to make dialysate. In contrast, ultrapure dialysate, commonly used in Europe but not widely in the US, has an ET level threshold of <0.03 EU/mL [12–14]. More recently, iron supplementation and bacterial translocation from the gastrointestinal (GI) tract has been proposed to significantly contribute to elevated serum ET concentrations in HD patients [10, 15]. Many Gram-negative bacteria are normal flora in the GI tract, and colonization by these organisms can lead to exposure and potential endotoxemia via bacterial translocation across the intestinal lumen and bacteriolysis induced by systemic antibiotics [16, 17]. 

Sevelamer is a large, cationic polymer phosphate binder that has been shown to bind free ET and is associated with reduction of serum ET concentrations in vitro and *in vivo* [18–20]. This review will discuss pharmacology, mechanisms of action, and data regarding the potential impact on nutritional status of this pleiotropic effect of sevelamer. 

## 2. Endotoxin: A Potent Inflammatory Stimulus

**Figure 1:**
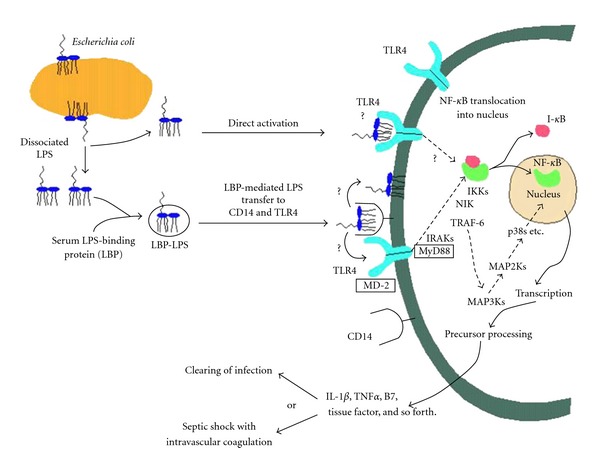
Lipopolysaccharide (endotoxin) signaling pathway. (Used with permission from [22].)

ET is a known inducer of systemic inflammation and endotoxemia and can induce negative cardiovascular effects and septic shock acutely [11]. The ET molecule consists of three structural domains including Lipid A, an oligosaccharide core and the distal O-antigen [21]. The distal O-antigen consists of a chain of common monosaccharide sugars and is structurally diverse dependent on the strain of bacteria. The inner oligosaccharide core is composed a negatively charged sugars that link the O-antigen to the Lipid A region. Lipid A is normally bound to the outer membrane in the cell wall of Gram-negative bacteria and is a negatively charged phospholipid structure. This is the active moiety that is responsible for recognition and binding to TLR4, which is mediated by LPS-binding protein (LBP) and CD14 (a coreceptor for LPS) that works in conjunction with TLR4 [22] (see Figure 1). The Proinflammatory pathway induced by ET activates neutrophils, dendritic cells, macrophages, and endothelial cells to release inflammatory cytokines, and if sustained can result in organ system failure and sudden death [23]. The pathway begins when ETs are recognized by LBP in addition to CD14, which escort LPS to the TLR4 and myeloid differentiation factor 2 (MD2) complex. This TLR4 and MD2 signaling complex is linked directly to a myeloid differentiation 88 (MyD88) and toll/interleukin-1 receptor domain containing adapter protein (TIRAP) signaling adaptor proteins that signal downstream enzymes to activate NF-*κ*B and AP1, that are translocated into the nucleus and lead to the release of inflammatory mediators (e.g., IL-1*β*, IL-6, and IL-18) [24]. These Proinflammatory cytokines are elevated in hemodialysis patients and have been associated with poor clinical outcomes, including increased risk of cardiovascular disease, hospitalization, and death [15, 25, 26]. 

Albumin has been shown to exhibit anti-inflammatory properties by binding to endotoxin and reducing the expression of pro-inflammation markers [27, 28]. Studies have explored the possibility of a correlation between decreased serum albumin concentrations and increased ET concentrations [10, 15]. Data from McIntyre et al. showed that lower serum albumin concentrations were associated with elevated serum ET; however, data by Feroze et al. did not corroborate this finding [10, 15]. The relationship between ET levels and other markers of poor nutritional status warrants further investigation.

## 3. Endotoxin Translocation in ESRD Patients

The GI tract is responsible for absorption of nutrients, and the mucosal surface acts as a protective intestinal barrier to bacteria, toxins, and microorganisms. Chronic inflammatory states including irritable bowel syndrome, heart failure, and chronic kidney disease (CKD) are associated with increases in gut permeability and bacterial translocation into the bloodstream, leading to endotoxemia [29]. The GI tract barrier can also be compromised by other conditions associated with ESRD including oxidative stress, circulatory compromise, hypoxia due to congestive heart failure, reduced gastrointestinal motility, and bacterial overgrowth [30]. It is hypothesized that the two most likely causes of increased serum ET levels in ESRD are gut hypoperfusion and bowel edema, which induces permeability changes and allows bacteria to translocate through the GI lumen into the bloodstream [31]. Iron is an essential growth factor for bacteria, and both oral and intravenous (IV) iron supplementation are common treatments for anemia in ESRD patients. Iron is an essential requirement for most microorganisms, and it has been shown that iron overload can enhance bacterial growth and virulence [32, 33]. 

It is well documented in the literature that patients receiving iron supplementation are at an increased risk of bacterial overgrowth and are more susceptible to bacterial translocation [34, 35]. Hepcidin is a polypeptide hormone that sequesters iron stores during acute inflammation to inhibit bacteria from acquiring iron in bacteremia [36]. In chronic inflammatory states such as ESRD, hepcidin inhibits intestinal uptake of iron increasing exposure locally. Thus, it is postulated that IV and oral iron could stimulate intestinal proliferation of bacteria potentially increasing bacterial translocation. IV iron products have also been shown to induce oxidative stress and hydroxyl free radical production in patients on hemodialysis that may reduce innate host defenses that would normally help to contain local bacterial loads [37]. 

## 4. Clinical Outcomes Associated with Elevated Endotoxin Levels

In an observational cohort study of 211 prevalent HD patients, Raj et al. showed that patients with serum soluble CD14 (indicative of increases in bound ET and ET exposure) concentrations in the highest tertile (>3.63 *μ*g/mL) were associated with an increased risk of all cause mortality (odds ratio 3.11; 95% CI 1.49–6.46; *P* = 0.002) compared to the referent low tertile group (<2.84 *μ*g/mL) [38]. In another cohort analysis, Feroze et al. observed that there was a positive correlation between serum ET levels and C-reactive protein (CRP); however, there was no statistically significant impact of ET on survival in unadjusted models or models adjusted for inflammation [15]. Correlation between serum ET concentration and CRP was also observed by Terawaki et al. [39]. Collectively, these data provide preliminary evidence that that low-grade endotoxemia may contribute to systemic inflammation in patients with ESRD. 

The inflammatory stimulus associated with elevated ET concentrations has been linked to increased cardiovascular disease risk with ESRD patients and may explain, in part, the markedly increased CVD death rate in hemodialysis patients [40]. Circulating ETs are associated with reduction in cardiac contractile performance and peripheral vasodilation [41]. In a cross-sectional study conducted by McIntyre et al., endotoxemia was found to be increased in more progressed stages of chronic kidney disease; however, there was no linear relationship to eGFR [10]. The highest serum ET concentrations were found in hemodialysis and peritoneal dialysis patients (mean ± SD for both dialysis modalities 0.62 ± 0.37 versus nondialysis patients 0.11 ± 0.68 EU/mL, *P* < 0.001). Pediatric hemodialysis patients also had markedly elevated ET serum concentrations compared to adult patients (mean 1.12 EU/mL, *P* = 0.008 versus adults). In multiple linear regression analyses, only CKD stage and serum albumin were independently associated with endotoxemia. In a univariate analysis, serum albumin was found to be negatively correlated with ET levels (*r* = −0.49, *P* < 0.001) [10]. 

## 5. Physicochemical Properties of Sevelamer

Sevelamer is a nonabsorbable hydrogel that does not contain aluminum, calcium, or magnesium in contrast to other available phosphate binding agents. The amine moiety includes a carbon backbone with multiple amine groups that are all separated from the carbon backbone by a single carbon [42]. These amine groups get protonated and bind to the anionic phosphate molecules in the intestine forming an insoluble complex that is excreted fecally. This decreases the overall absorption of dietary phosphate and decreases serum phosphorus levels [43]. It is orally administered and is excreted without any systemic absorption during its transport through the gastrointestinal tract. Sevelamer carbonate and sevelamer hydrochloride have the same polymeric structure with the major difference being that the carbonate replaced all of the chloride counter ions in the hydrochloride salt product [44, 45]. Sevelamer has been found to bind to bile acids in vitro and *in vivo* probably due to its physiochemical similarities to common bile sequestrants, thereby interfering with fat absorption and reducing low-density lipoprotein (LDL) cholesterol levels [46]. Sevelamer carbonate (Renvela) is a carbonate buffered amine that binds to phosphate and other anions as a resin [43]. Sevelamer carbonate is approved for use in CKD patients on dialysis and has gradually replaced sevelamer hydrochloride (Renagel) due to its ability to donate base equivalents and contribute to treatment of chronic metabolic acidosis in ESRD patients [47].

## 6. Sevelamer Binding of Endotoxin

Sevelamer can nonspecifically bind to other negatively charged biomolecules [18]. Sevelamer is known to bind negatively charged bile acids, thus acting as a bile acid sequestrant that can lower low-density lipoprotein concentrations [43]. The negatively charged Lipid A portion of ET allows for binding to sevelamer. An in vitro study investigated the ET binding affinity of sevelamer hydrochloride using 10 ng/mL of *E*. *coli* derived ET across a range of sevelamer hydrochloride concentrations (0–50 mg/mL) at time points ranging from 0.5 to 24 hours. Free ET was measured by the limulus amebocyte assay. A range of pH conditions were studied to simulate various GI environments. A reduction of free ET was observed at each time point across all dose ranges and exhibited a dose-dependent response. The one hour time point was noted to be the free ET nadir for the 5, 10, 20, and 50 mg/L doses. There was no difference in ET binding affinity by sevelamer across pH ranges. Interestingly, as the phosphate concentration was increased in the binding affinity experiments, the authors showed increased ET binding in a dose-dependent manner. This suggests a cooperative binding effect, that has also been suggested from previous studies that have shown sevelamer can lose significant binding affinity for phosphate in the presence of bile acids [48]. Finally, the authors used a monocyte cell culture model to evaluate tumor necrosis factor-alpha (TNF-*α*) and subsequent reduction with cotreatment with ET and sevelamer. A dose-dependent reduction in cell supernatant TNF-*α* concentrations that was observed in cells treated with sevelamer indicated the ET binding properties affect downstream inflammation pathways [18]. In a translational study investigating the effect of sevelamer on ET concentrations *in vivo*, a rat model of renal failure was created by performing a 5/6 nephrectomy. After nephrectomy, plasma ET concentrations increased significantly in the uremic control animals (not treated with sevelamer) versus sham controls, indicating the uremic state itself is associated with increased GI permeability to ET. Animals were randomized to one of four groups: uremic rats treated with sevelamer 3% added to chow, uremic rats not treated with sevelamer, sham rats treated with sevelamer 3% added to chow, and sham rats not treated with sevelamer and followed for 60 days. The uremic sevelamer-treated rats had significantly lower ET and TNF-*α* concentration at all time points: days 7, 30, and 60 compared to uremic control rats. Sevelamer treatment appeared to mitigate the rise in C-reactive protein (CRP) in the uremic rats; however, this did not reach statistical significance. Both sham rat groups had no increase in ET and undetectable TNF-*α* concentrations [26]. 

## 7. Clinical Trials of Endotoxin Binding by Sevelamer

Recent clinical and nonclinical studies of the effects of sevelamer on free ET and Proinflammatory markers are summarized in Table 1. An observational cross-sectional study that included 46 maintenance hemodialysis patients, collected predialysis blood samples prior to two consecutive dialysis and measured plasma ET, IL-6, and CRP. Mean plasma ET was lower in patients using sevelamer (*n* = 30) [19]. In multivariate regression analyses, sevelamer use independently predicted lower plasma ET after adjustment for multiple confounders including race, gender, age, dialysis vintage, total cholesterol, and white blood cell count. Binder dose (mean ± SD; 3,461 ± 505 mg/day) and duration of treatment were not predictors of ET levels. There was also no correlation between ET concentration and plasma IL-6 or CRP identified in this study. In a more recent prospective, randomized, and open label study, 59 hemodialysis patients were randomized to sevelamer hydrochloride (*n* = 30, Renagel 1600 mg three times a day) or calcium acetate (*n* = 29, Royen 500 mg three times a day) for twelve weeks [49]. Serum concentrations of ET, CRP, soluble CD14, Proinflammatory cytokines (TNF-*α*, IL-1, IL-6, and IL-18) and an anti-inflammatory cytokine (IL-10) were measured at baseline and end of study. Serum ET and soluble CD14 were significantly reduced in sevelamer treated patients (22.6% and 15.2%, resp., *P* < 0.01 for both analyses). The only Proinflammatory cytokine that significantly decreased among sevelamer treated patients was IL-6 by approximately 7% (*P* < 0.0001 versus baseline values). The anti-inflammatory cytokine IL-10, increased by approximately 15% from baseline (*P* = 0.052). CRP also decreased from baseline in the sevelamer group. There were no significant changes from baseline in any measures in the calcium acetate group [49].

## 8. Summary

Elevated ET concentrations are associated with systemic inflammation, poor nutritional status, cardiovascular disease, and increased mortality risk in patients with CKD [10]. Increased systemic ET in HD patients is presumably due to dialysate exposure, translocation of ET from the gut secondary to gut permeability changes and possibly enhanced by iron product use. Sevelamer is physicochemically capable of binding the negatively charged Lipid A portion of ET, and in vitro experiments demonstrate ET binding that is dose-dependent [18]. An additive secondary mechanism of ET reduction by sevelamer may be an indirect binding mechanism that is associated with its known bile acid binding characteristics. Several small, short-term studies have shown an association between sevelamer treatment and decreases in ET, soluble CD14, and Proinflammatory markers, such as CRP and IL-6, biomarkers that have been strongly linked to increased mortality rates in the CKD population [19, 20, 25, 49]. The marked 78% reduction of CRP levels in hemodialysis patients compared to baseline in the study by Stinghen et al. may indicate that there are additional Proinflammatory compounds that could be bound by sevelamer. A recent 8-week randomized, crossover study in CKD patents at stages from 2 to 4 demonstrated that sevelamer carbonate treatment reduced advanced glycation end products, hemoglobin A1C, and biomarkers of inflammation (8 isoprostanes and monocyte intracellular TNF-*α*) indicating there may be important clinical implications of binding other Proinflammatory mediators [50]. These preliminary data suggest potential benefits of sevelamer in reducing Proinflammatory cytokines especially in patients in early diabetic CKD; however, significantly more research is warranted. A large randomized controlled trial of sevelamer HCl versus calcium acetate did not show any survival benefit in maintenance HD patients randomized to sevelamer [51]. This is in contrast, however, to a recently published randomized trial in CKD stage 3 and 4 randomized patients to sevelamer or calcium carbonate and followed patients for 36 months [52]. In these earlier-stage patients sevelamer treatment was associated with a reduced risk of all cause mortality (hazard ratio = 0.36 95%, CI 0.15–0.83). These data suggest that survival benefits from interventions to reduce inflammation could potentially be realized among predialysis patients [52]. 

In summary, there are compelling preliminary data that sevelamer effectively binds ET and preliminary data that suggest effective binding of ET by sevelamer with associated decreases in robust biomarkers of inflammation that have been strongly linked malnutrition and mortality in ESRD. Additional studies are needed to determine if improvement in clinical outcomes could be realized with sevelamer treatment in HD patients with poor nutritional status and elevated ET.

## Figures and Tables

**Table 1:** Recent interventional trials of sevelamer and impact on proinflammatory biomarkers.

Authors	Study design	Treatment groups	Outcomes/results	*P* value
Hauser et al., 2010 [25]	Nonclinical animal model (Rats surgical nephrectomy-induced uremia)	Rats fed *normal* chow (*n* = 7) or 3% *sevelamer carbonate* added to chow for 60 days (*n* = 5) (Plasma levels of TNF-*α*, ET, and CRP measured at 7, 30, and 60 days)	Plasma levels were reduced in sevelamer-treated rats versus control uremic rats: TNF-*α* ET *There was no significant change in CRP levels *	*<0.05* *<0.005 *

Sun et al., 2009 [19]	Cross-sectional study of 46 stable HD patients	Sevelamer HCl (*n* = 30) or nonsevelamer (*n* = 16) (CRP, IL-6, and ET levels (at two consecutive blood samples during dialysis))	Plasma levels in patients on sevelamer versus not on sevelamer: ET: 0.23 ± 0.01 versus 0.3 ± 0.01 EU/mL IL-6: 9.6 ± 1.2 versus 10.1 ± 1.7 pg/mL CRP: 16.1 ± 1.2 versus 15.6 ± 2.2 mcg/mL	*0.001* * 0.927* * 0.892 *

Stinghen et al., 2010 [20]	Prospective study in stable 26 HD patients eligible for switch to sevelamer (per KDOQI guidelines)	All (*n* = 26) patients started on sevelamer HCl plasma *hs*CRP and ET measured; at Baseline and at 6 months	Plasma concentrations of *hs*CRP and ET decreased from baseline, mean (SD):^‡^ *hs*CRP: 4.8 ± 1.2 to 0.44 ± 0.12 mg/L ET: 3.6 ± 0.8 to 1.2 ± 0.6 EU/mL	*<0.001* *<0.005 *

Navarro-González et al., 2011 [49]	Prospective, randomized, open-label, and parallel design trial in stable HD patients (*N* = 59)	Sevelamer HCl (*n* = 30) or calcium acetate (*n* = 29) (CRP, TNF-*α*, IL-1, IL-6, IL-10, IL-18, sCD14, and ET serum levels measured at baseline and 3 months after therapy)	Serum levels in the sevelamer group at *baseline* versus at *3 month followup*; median (interquartile range):^†^ ET: 0.58 (0.51–0.60) down to 0.42 (0.27–0.54) EU/mL CRP: 6.9 (5.9–8) down to 5.9 (5.1–10) mg/mL IL-6: 5 (3–9.1) down to 4.9 (2.68–8.6) pg/mL	*0.001* *0.001* *0.01 *

Vlassara et al., 2012 [50]	Single-center, randomized, 2-month, open-label, intention-to-treat, and *crossover* study in patients with DM-2 stage 2–4 CKD (*N* = 20)	Sevelamer carbonate versus calcium carbonate HbA1c, FGF-23, TNF-*α*, and *hs*CRP measured after 8 weeks on each treatment modality (separated by 1 week wash-out period)	Estimated effect of sevelamer relative to calcium carbonate (after adjusting for period of treatment):^¶^ HbA1c: −0.67 (95% CI, from −1.25 to −0.10) FGF-23: −59.74 (95% CI, from −116.35 to −3.1) TNF-*α*: −7.2 (95% CI, from −11.01 to −3.38)	0.02 0.04 <0.001

HD: hemodialysis; TNF-*α*: tumor necrosis factor; IL: interleukin; sCD: *soluble* cluster differentiation; ET: endotoxin; *hs*CRP: *high sensitivity* C-reactive protein; FGF-23: fibroblast growth factor-23; HbA1c: hemoglobin A1c; CKD: chronic kidney disease, KDOQI: national kidney foundation disease outcomes quality initiative; DM-2: diabetes mellitus type-2.

^†^sCD14 also showed significant change from baseline in the sevelamer groups. No significant change in the calcium acetate group or in the other outcomes.

^‡^No control group.

^¶^Effect on *hs*CRP levels did not differ significantly between the groups.

## References

[1] Stenvinkel P, Alvestrand A (2002). Inflammation in end-stage renal disease: sources, consequences, and therapy. *Seminars in Dialysis*.

[2] Ikizler TA, Wingard RL, Harvell J, Shyr Y, Hakim RM (1999). Association of morbidity with markers of nutrition and inflammation in chronic hemodialysis patients: a prospective study. *Kidney International*.

[3] Carrero JJ, Qureshi AR, Axelsson J (2007). Comparison of nutritional and inflammatory markers in dialysis patients with reduced appetite. *The American Journal of Clinical Nutrition*.

[4] Don BR, Kaysen G (2004). Serum albumin: relationship to inflammation and nutrition. *Seminars in Dialysis*.

[5] Raj DS, Carrero JJ, Shah VO (2009). Soluble CD14 Levels, Interleukin 6, and mortality among prevalent hemodialysis patients. *The American Journal of Kidney Diseases*.

[6] Kalantar-Zadeh K, Block G, McAllister CJ, Humphreys MH, Kopple JD (2004). Appetite and inflammation, nutrition, anemia, and clinical outcome in hemodialysis patients. *The American Journal of Clinical Nutrition*.

[7] Ikizler TA, Pupim LB, Brouillette JR (2002). Hemodialysis stimulates muscle and whole body protein loss and alters substrate oxidation. *The American Journal of Physiology*.

[8] Vannini FD, Antunes AA, Caramori JCT, Martin LC, Barretti P (2009). Associations between nutritional markers and inflammation in hemodialysis patients. *International Urology and Nephrology*.

[9] Anker SD, Ponikowski P, Varney S (1997). Wasting as independent risk factor for mortality in chronic heart failure. *The Lancet*.

[10] McIntyre CW, Harrison LEA, Eldehni MT (2011). Circulating endotoxemia: a novel factor in systemic inflammation and cardiovascular disease in chronic kidney disease. *Clinical Journal of the American Society of Nephrology*.

[11] Beutler B, Rietschel ET (2003). Innate immune sensing and its roots: the story of endotoxin. *Nature Reviews Immunology*.

[12] Ward RA (2011). New AAMI standards for dialysis fluids. *Nephrology News & Issues*.

[13] Arizono K, Nomura K, Motoyama T (2004). Use of ultrapure dialysate in reduction of chronic inflammation during hemodialysis. *Blood Purification*.

[14] Hsu PY, Lin CL, Yu CC (2004). Ultrapure dialysate improves iron utilization and erythropoietin response in chronic hemodialysis patients: a prospective cross-over study. *Journal of Nephrology*.

[15] Feroze U, Kalantar-Zadeh K, Sterling KA (2012). Examining associations of circulating endotoxin with nutritional status, inflammation, and mortality in hemodialysis patients. *Journal of Renal Nutrition*.

[16] Genth-Zotz S, von Haehling S, Bolger AP (2002). Pathophysiologic quantities of endotoxin-induced tumor necrosis factor-alpha release in whole blood from patients with chronic heart failure. *The American Journal of Cardiology*.

[17] Gonclaves S, Pecoits-Filho R, Perreto S (2005). Fluid overload is associated with endotoxinemia but not with systemic inflammation in chronic kidney disease patients. *Journal of the American Society of Nephrology*.

[18] Perianayagam MC, Jaber BL (2008). Endotoxin-binding affinity of sevelamer hydrochloride. *The American Journal of Nephrology*.

[19] Sun PP, Perianayagam MC, Jaber BL (2009). Sevelamer hydrochloride use and circulating endotoxin in hemodialysis patients: a pilot cross-sectional study. *Journal of Renal Nutrition*.

[20] Stinghen AEM, Gonçalves SM, Bucharles S (2010). Sevelamer decreases systemic inflammation in parallel to a reduction in endotoxemia. *Blood Purification*.

[21] van Amersfoort ES, van Berkel TJC, Kuiper J (2003). Receptors, mediators, and mechanisms involved in bacterial sepsis and septic shock. *Clinical Microbiology Reviews*.

[22] Raetz CRH, Whitfield C (2002). Lipopolysaccharide endotoxins. *Annual Review of Biochemistry*.

[23] Zanoni I, Ostuni R, Capuano G (2009). CD14 regulates the dendritic cell life cycle after LPS exposure through NFAT activation. *Nature*.

[24] Zanoni I, Ostuni R, Marek LR (2011). CD14 controls the LPS-induced endocytosis of toll-like receptor 4. *Cell*.

[25] Hauser AB, Azevedo IRF, Gonçalves S, Stinghen A, Aita C, Pecoits-Filho R (2010). Sevelamer carbonate reduces inflammation and endotoxemia in an animal model of uremia. *Blood Purification*.

[26] Noori N, Kovesdy CP, Dukkipati R (2011). Racial and ethnic differences in mortality of hemodialysis patients: role of dietary and nutritional status and inflammation. *The American Journal of Nephrology*.

[27] Powers KA, Kapus A, Khadaroo RG, Papia G, Rotstein OD (2002). 25% albumin modulates adhesive interactions between neutrophils and the endothelium following shock/resuscitation. *Surgery*.

[28] Kitano H, Fukui H, Okamoto Y (1996). Role of albumin and high-density lipoprotein as endotoxin-binding proteins in rats with acute and chronic alcohol loading. *Alcoholism Clinical and Experimental Research*.

[29] Kotanko P, Carter M, Levin NW (2006). Intestinal bacterial microflora—a potential source of chronic inflammation in patients with chronic kidney disease. *Nephrology Dialysis Transplantation*.

[30] Ding LA, Li JS (2003). Gut in diseases: physiological elements and their clinical significance. *World Journal of Gastroenterology*.

[31] Krack A, Sharma R, Figulla HR, Anker SD (2005). The importance of the gastrointestinal system in the pathogenesis of heart failure. *European Heart Journal*.

[32] Bullen JJ, Rogers HJ, Spalding PB, Ward CG (2005). Iron and infection: the heart of the matter. *FEMS Immunology and Medical Microbiology*.

[33] Boelaert JR, Daniels RF, Schurgers ML, Matthys EG, Gordts BZ, van Landuyt HW (1990). Iron overload in haemodialysis patients increases the risk of bacteraemia: a prospective study. *Nephrology Dialysis Transplantation*.

[34] Walter T, Olivares M, Pizarro F, Muñoz C (1997). Iron, anemia, and infection. *Nutrition Reviews*.

[35] Ashrafian H (2003). Hepcidin: the missing link between hemochromatosis and infections. *Infection and Immunity*.

[36] Robson KJ (2004). Hepcidin and its role in iron absorption. *Gut*.

[37] Pai AB, Depczynski J, Pai MP, McQuade CR, Mercier RC (2006). Non-transferrin-bound iron is associated with enhanced *Staphylococcus aureus* growth in hemodialysis patients receiving intravenous iron sucrose. *The American Journal of Nephrology*.

[38] Raj DSC, Shah VO, Rambod M, Kovesdy CP, Kalantar-Zadeh K (2009). Association of soluble endotoxin receptor CD14 and mortality among patients undergoing hemodialysis. *The American Journal of Kidney Diseases*.

[39] Terawaki H, Yokoyama K, Yamada Y (2010). Low-grade endotoxemia contributes to chronic inflammation in hemodialysis patients: examination with a novel lipopolysaccharide detection method. *Therapeutic Apheresis and Dialysis*.

[40] Wanner C, Drechsler C, Krane V (2009). C-reactive protein and uremia. *Seminars in Dialysis*.

[41] Kumar A, Haery C, Parrillo JE (2000). Myocardial dysfunction in septic shock. *Critical Care Clinics*.

[42] Wrong O, Harland C (2007). Sevelamer and other anion-exchange resins in the prevention and treatment of hyperphosphataemia in chronic renal failure. *Nephron Physiology*.

[43] Barna MM, Kapoian T, O’Mara NB (2010). Sevelamer carbonate. *Annals of Pharmacotherapy*.

[44] Genzyme Corporation (2011). *Renagel (Sevelamer Hydrochloride) Package Insert*.

[45] Genzyme Corporation (2011). *Renvela (Sevelamer Carbonate) Package Insert*.

[46] Braunlin W, Zhorov E, Guo A (2002). Bile acid binding to sevelamer HCl. *Kidney International*.

[47] Pai AB, Shepler BM (2009). Comparison of sevelamer hydrochloride and sevelamer carbonate: risk of metabolic acidosis and clinical implications. *Pharmacotherapy*.

[48] Autissier V, Damment SJP, Henderson RA (2007). Relative in vitro efficacy of the phosphate binders lanthanum carbonate and sevelamer hydrochloride. *Journal of Pharmaceutical Sciences*.

[49] Navarro-González JF, Mora-Fernández C, de Fuentes MM, Donate-Correa J, Cazaña-Pérez V, García-Pérez J (2011). Effect of phosphate binders on serum inflammatory profile, soluble CD14, and endotoxin levels in hemodialysis patients. *Clinical Journal of the American Society of Nephrology*.

[50] Vlassara H, Uribarri J, Cai W (2012). Effects of sevelamer on HbA1c, inflammation, and advanced glycation end products in diabetic kidney disease. *Clinical Journal of the American Society of Nephrology*.

[51] Suki WN (2008). Effects of sevelamer and calcium-based phosphate binders on mortality in hemodialysis patients: results of a randomized clinical trial. *Journal of Renal Nutrition*.

[52] Di Iorio B, Bellasi A, Russo D (2012). Mortality in kidney disease patients treated with phosphate binders: a randomized study. *Clinical Journal of the American Society of Nephrology*.

